# Perceived stress across population segments characterized by differing stressor profiles—A latent class analysis

**DOI:** 10.1371/journal.pone.0316759

**Published:** 2025-01-16

**Authors:** Finn Breinholt Larsen, Mathias Lasgaard, Morten Vejs Willert, Jes Bak Sørensen

**Affiliations:** 1 DEFACTUM, Central Denmark Region, Aarhus, Denmark; 2 Department of Psychology, University of Southern Denmark, Odense, Denmark; 3 Department of Occupational Medicine, Danish Ramazzini Centre, Aarhus University Hospital, Aarhus, Denmark; 4 AIAS, Aarhus Institute of Advanced Studies, Aarhus University, Aarhus, Denmark; The John Paul II Catholic University of Lublin, POLAND

## Abstract

**Objective:**

We aimed to 1) identify distinct segments within the general population characterized by various combinations of stressors (stressor profiles) and to 2) examine the socio-demographic composition of these segments and their associations with perceived stress levels.

**Methods:**

Segmentation was carried out by latent class analysis of nine self-reported stressors in a representative sample of Danish adults (N = 32,417) aged 16+ years. Perceived stress level was measured by the Perceived Stress Scale (PSS).

**Results:**

Seven classes were identified: Class 1 was labeled Low Stressor Burden (64% of the population) and the remaining six classes, which had different stressor combinations, were labeled: 2) Burdened by Financial, Work, and Housing Stressors (10%); 3) Burdened by Disease and Death among Close Relatives (9%); 4) Burdened by Poor Social Support and Strained Relationships (8%); 5) Burdened by Own Disease (6%); 6) Complex Stressor Burden Involving Financial, Work, and Housing Stressors (2%); and 7) Complex Stressor Burden Involving Own Disease and Disease and Death among Close Relatives (2%). Being female notably increased the likelihood of belonging to Classes 2, 3, 5, and 7. Higher age increased the likelihood of belonging to Class 3. Low educational attainment increased the likelihood of belonging to Classes 5 and 6. A significant difference was observed in perceived stress levels between the seven latent classes. Average PSS varied from 9.0 in Class 1 to 24.2 in Class 7 and 25.0 in Class 6.

**Conclusion:**

Latent class analysis allowed us to identify seven population segments with various stressor combinations. Six of the segments had elevated perceived stress levels but differed in terms of socioeconomic composition and stressor combinations. These insights may inform a strategy aimed at improving mental health in the general population by targeting efforts to particular population segments, notably segments experiencing challenging life situations.

## Introduction

Stress is a major cause of psychological distress and disease, making it a significant public health issue [1]. Recent studies indicated that the majority of the general population continues to experience low levels of perceived stress [2–5]. However, a concerning rise was recorded in the proportion of individuals encountering high levels of perceived stress, particularly among younger generations [5, 6]. This trend is likely exacerbated by the negative impact of the COVID-19 pandemic on population mental health and well-being [7].

This situation warrants intensified population level efforts. An effective public health strategy to counter stress should be based on knowledge of 1) the main sources of stress in contemporary societies and 2) how sources of stress cluster and affect stress levels in segments of the population with various stressor profiles. Below, stressors are understood as any physical, psychological, or social event or condition that may cause stress in individuals and ultimately challenge physical health and mental well-being [8].

Previously, we used a variable-centered approach to explore the main sources of stress in contemporary societies. Our findings showed that among a general population own disease, work situation, and poor social support were the primary sources of perceived stress among a wide range of work-related and non-work-related stressors [9, 10]. However, the results also testified to the need for a comprehensive approach to stress since most stressors had an effect on perceived stress level [10]. In this study, we examined the second topic by adopting a person-centered approach to identify distinct groups of individuals within the general population who share similar stressor patterns. Specifically, we examined the size of each population segment, the characteristics of their stressor profiles, the socio-demographic composition of the segments, and their associations with perceived stress levels. We paid particular attention to identifying general population segments in which the combination of stressors was associated with high stress levels, highlighting a need for interventions targeting these groups.

Consistent with our previous studies, the transactional stress model (TSM), proposed by Lazarus and Folkman [11], and the stress process model (SPM), proposed by Pearlin [12], provided the theoretical backdrop of the study. In conjunction, the two theories support a perception of stress as a psychological, but socially generated and structured, phenomenon [9]. According to the TSM, stress arises when a situation is perceived as threatening or challenging and demands strain or exceeds the individual’s ability to cope adequately. The SPM claims that stress results from a social process where stressors are distributed between groups and individuals on the basis of social characteristics. Both theories leave it to empirical investigation to determine which stressors are the primary sources of stress and how stressors cluster in certain groups and individuals across populations.

More recent theoretical developments have added to our understanding of the importance of the combined effects of multiple stressors on an individual’s well-being. *Stressor diversity* refers to the extent to which stressor events are spread across multiple domains of life, such as work-related stress, family conflicts, financial pressures, and health issues [13, 14]. Research has shown that experiencing greater stressor diversity is linked to better affective well-being than experiencing the same amount of stressors within a single domain [13]. The *overpowering hypothesis* predicts that older adults will respond more strongly than younger people to unpleasant events affecting multiple life domains (complex events) [15]. The *multiple burden hypothesis* predicts that combining a paid job, being married, and having children is likely to have negative effects on women’s health [16]. The *role overload* model proposes that individuals who need to balance the demands and obligations of different social roles may generate "overloads" that prejudice their health [17]. The *stress proliferation* theory posits that stress in one domain of a person’s life may result in stress "spillover", affecting other domains [18].

In conjunction, these theories highlight the complex nature of stress and the importance of considering multiple stressors potentially impacting an individual’s well-being. They also suggest that interventions aimed at reducing stress should take into account the diverse range of stressors that people may be facing and any potential interactions between them.

Various research methods are available for person-oriented research [19]. One is *latent class analysis* (LCA), which we used in this study. LCA is a statistical method used to identify unobserved subgroups within a population [20]. LCA rests on the assumption that the observed relationships between a group of indicators can be explained by the existence of an unobserved categorical latent variable with K categories where each category represents a latent class. When the population is divided into subgroups based on the latent variable, the indicators within each subgroup become statistically independent (local independence assumption). In other words, the subgroups will be internally homogeneous with respect to the observed indicators but differ from other subgroups. The main output from a LCA is the number and size of latent classes and the response probability of each indicator given that a person belongs to a certain class. In this study, LCA allowed us to identify distinct population segments with distinct stressor profiles.

LCA is based on a stochastic model and individuals are not deterministically assigned to a latent class. Instead, an individual belongs to each of the K latent classes with a probability ranging from 0 to 1. Consider two individuals who are both more likely to belong to class k1 than to one of the other classes and that the first person has a higher probability of belonging to the class than the second person. In this case, the first person’s response pattern can be considered more "typical" of that class than the second person’s response pattern [21].

LCA was used to examine the clustering of occupational stressors and their association with background characteristics and work-related outcomes, including mental health and self-reported stress [22–32]. Furthermore, LCA has been used to identify patterns of stress exposures within certain population segments. For instance, it was used to examine stress patterns among older black Americans and explore how these patterns were related to depressive symptoms [33]; and among youth in residential care to investigate their association with major psychiatric problems [34]. However, to the best of our knowledge, the present study is the first to examine the clustering of stressors and their association with perceived stress in a general population.

## Methods

### Study design and data collection

We used data from the 2017 health survey “How are you?”, implemented by the Central Denmark Region. “How are you?” is a representative, cross-sectional survey of the population aged 16 years and above. It is based on a stratified random sample of people living in the Central Denmark Region as per 1 January 2017. The sample was drawn from the Danish Population Register. A total of 52,000 people were invited to participate. Participants were invited to complete a web-based or postal questionnaire [35]. Data collection started on February 3rd 2017 and ended on May 3rd 2017. Those who failed to respond to the invitation received up to four reminders. The response rate was 62% (32,417), and 80% of the respondents completed the web questionnaire. The study was approved by the Danish Data Protection Agency (r. no. 2012-58-0006) and the Central Denmark Region (r. no. 1–16–02-593-16). Danish law requires no formal ethical approval of survey and register-based studies from an ethics committee or other research oversight (§ 14 section 2) [36]. Furthermore, Danish law does not require consent from parents or guardians for the participation of minors above the age of 15 in surveys. Each participant received written information about the purpose of the study and was informed that by answering the questionnaire, they consented to participate. All parts of the survey were implemented in accordance with relevant guidelines and regulations along with the approval. Data from the survey were linked with national administrative registers using a unique personal identification number assigned to all Danish citizens [37]. Register data included age, gender, and ethnic background. To reduce sampling and non-response bias, we applied weights constructed by Statistics Denmark using a model-based calibration approach and including socio-demographic characteristics, income, social benefits, and healthcare utilization in both the descriptive and model-based analyses [35, 38].

### Variables

The questions used to assess perceived stressors cover major life events, chronic stressors, and daily hassles and were adapted from the Life Event Questionnaire [39]. The set of questions was originally selected for the Danish National Birth Cohort [40–42] and included in "How are you?", with a few modifications. The initial question was: “Within the past 12 months, have you felt burdened by some of the following things?” The questionnaire covers work situation, financial circumstances, housing conditions, relationship with partner, relationship with family and friends, own disease, disease among close relatives, and deaths among close relatives. The response categories were “no”, “yes, a little”, “yes, partly” or “yes, a lot”. In the present study, the four response categories were dichotomized into "no/a little" and "partly/a lot". Another potential stressor, lack of perceived social support, was assessed using the following question: "Do you have someone to talk to if you have problems or need support?" The response format was “yes, always”, “yes, most of the time”, “yes, sometimes” or “no, never or almost never”. Here, we dichotomized the response categories into "always/mostly" and "sometimes/never or almost never".

Age and sex were assessed using a combination of self-reported and register data. Ethnic background was defined using the Danish Civil Registration System [37]. Educational attainment was self-reported and categorized as low (0–10 years), medium (11–15 years), or high (15+ years) according to the Danish version of the International Standard Classification of Education [43]. Students were categorized according to their expected graduation level. Self-reported data were used to assess work status, cohabitation status, and whether or not the participants lived with children aged 0–15 years.

Level of perceived stress was assessed by the 10-item Perceived Stress Scale (PSS) [44]. Based on Lazarus’ stress model [45, 46], the PSS is a global stress measure for the extent to which events or situations are perceived as demanding or threatening. The ten items ask how often in the past month life was appraised as unpredictable, uncontrollable, and overloaded (e.g., “In the last month, how often have you been upset because of something that happened unexpectedly?”) [44]. The items were scored from 0 to 4 (“never”, “almost never”, “sometimes”, “fairly often”, or “very often”). An increasing sum score (range 0 to 40) indicates an increasing perceived stress level [44]. PSS does not have validated cut-off values for determining high or low levels of perceived stress [44]. However, a number of studies have proposed cut-off values ranging from 15 to 18 for high levels of perceived stress [47–50]. PSS has satisfying psychometric properties [51, 52] and has been used in a number of population-based studies. In the present study, Cronbach’s α indicated that the scale had a high internal consistency (α = 0.87). We report PSS as a continuous variable and as a dichotomized variable with a cut-off of 18.

Before conducting the analyses, data were screened for missing values. The proportion of missing values on perceived stressors varied between 6.3% (lack of perceived social support) and 9.8% (work situation). For the eight items adapted from the Life Event Questionnaire, missing values were treated as “no” if respondents had answered at least one question. Consequently, 28,201 observations (87%) had complete data for all eight items. Imputation was performed for 3462 observations (6%) with one to seven missing responses, while 2053 observations (7%) with missing values across all eight variables remained unchanged. The social support question had 2177 missing responses (7%). In total, there were 30.133 observations with complete data on the nine perceived stressor items after imputation.

Missing data for the PSS items ranged from 4.3% (item 1) to 5.1% (item 8). When fewer than four out of ten PSS items were missing, the mean of the available items was used to compute the scale score. If four or more items were missing, the PSS score was considered missing. This approach resulted in PSS scores for 29,476 observations (91%) with complete data for all ten items, and for 1549 observations (4.8%) with one to three missing items, while 1392 observations (4.3%) did not receive a PSS score.

The LCA and subsequent analyses were conducted using complete cases. The results were compared to those derived from the full sample (32,417) using full information maximum likelihood (FIML), which managed missing data directly in the likelihood function. No substantial differences were noted in cluster size or indicator prevalence, but models based on FIML showed poorer relative fit and lower entropy values compared to models based on complete cases (0.53 with FIML versus 0.59 for complete cases)

### LCA approach

The model-based analysis in this study comprised three steps: (1) segments of the general population with different stressor profiles were identified using LCA; (2) the socio-demographic composition of the segments was examined; and (3) the relationship between stressor profiles and perceived stress was analyzed. All analyses were conducted using Latent GOLD 6.0.

In the first step, we estimated LCA models that had between one and ten latent classes. The final model was selected based on statistical criteria (optimal fit) and interpretability. For model comparison, we used the Akaike Information Criterion (AIC) and the Bayesian Information Criterion (BIC). Both are measures of relative fit and seek to balance model complexity against sample size. In both measures, a lower value indicates a better model fit. The BIC prefers simpler models (fewer classes) than the AIC [53]. BIC is generally considered the more reliable of the two measures when the sample size is large as it protects against overfitting [54]. Entropy was used to describe the ability of the selected model to uniquely assign individuals to a certain class (class separation), where a high value equals good class separation. LCA rests on the assumption of local independence between indicators. Bivariate residuals (BVR) were examined as a means of describing possible sources of misfit in the selected model. A high BVR for a pair of variables indicates residual local dependency that causes model misfit. If theoretically justified, a model can be modified to account for local dependence between two variables.

In the second step, the sociodemographic composition of the stressor segments found in the first step was described. The description included the distribution of each group by age, sex, ethnic origin, educational attainment, work status, cohabitation status, and children in the home. A multinomial logistic regression model was used to predict how the individual variable affected the probability of belonging to the different stressor groups when adjusting for the other covariates.

In the third step, we examined the variation in perceived stress across stressor segments using one-way analysis of variance (ANOVA). For the second and third steps, we used procedures developed in recent years to relate latent class membership to external variables that adjust for the fact that each subject is assigned to a latent class with less than 100% certainty, thereby reducing bias in the estimates due to classification uncertainty [55–58]. For the bivariate analysis of the sociodemographic composition of the stressor segments and the multivariate multinomial logistic regression model, we applied the two-step correction method outlined by Bakk and Kuha [58]. In the first step, the selected LCA model was estimated without incorporating external variables. In the second step, the parameters of the LCA model were fixed at their estimated values and the relationship between the covariates and the latent classes was assessed. The two-step method is particularly effective for minimizing bias from classification uncertainty in models that includes covariates, especially when the LCA measurement model exhibits poor separation between latent classes (low entropy).

For the ANOVA model, we employed the three-step method with the correction procedure initially introduced by Bolck et al. [56] and later refined by Bakk et al. [57]. In the first step, the chosen latent class model was estimated without external variables. In the second step, each respondent was assigned to their most likely latent class. The third step involved estimating the relationship between the latent classes and the distal outcome (PSS), while adjusting for bias due to classification uncertainty.

## Results

### Sample description

Table 1 describes the distribution of the study sample. The mean age was 47.6 years (standard deviation (SD) 19.3 years, range 16–102 years), with an equal distribution of men and women. High educational attainment was reported by 29% of the participants, 59% were employed, and 66% were married or cohabitating, while 28% were residing with at least one child aged 0–15 years. Nine out of ten sampled people were of Danish ethnic origin. The most commonly reported stressors were related to work, own disease, and financial circumstances, whereas stressors related to relationships with family or friends were less prevalent.

**Table 1:** Characteristics of study population.

Characteristics	N	Weighted prevalence (%)
Sex		
Male	15,105	50
Female	17,312	50
Age (mean (SD))		47.6 (19.3)
Age (years)		
16–24	3,790	15
25–34	3,423	15
35–44	4,619	15
45–54	5,914	17
55–64	5,845	15
65–74	5,702	14
75+	3,124	9
Educational attainment		
Low (0–10 years)	4,580	16
Medium (11–15 years)	17,050	54
High (15- years)	8,518	29
Cohabitation status		
Married/cohabitating	22,314	66
Single	8,396	34
Ethnic origin		
Danish	30,469	89
Other Western	847	4
Non-Western	1,101	6
Work status		
Working	17,775	59
Non-working	12,218	41
Living with child(ren) aged 0–15 years		
No	20,145	72
Yes	7,452	28
Burdened by the following stressors within the last 12 months (partly/a lot)		
Financial circumstances	4,020	16
Housing conditions	2,117	9
Work situation	4,748	17
Relationship with partner	2,134	8
Relationship with family and friends	1,587	6
Disease	5,223	17
Disease among close relatives	4,277	14
Deaths among close relatives	2,445	8
Lack of perceived social support	4,159	15
Number of reported perceived stressors (mean (SD))		1.10 (1.45)

SD = standard deviation

### Selection of latent class model

Table 2 summarizes the fit criteria for latent class models with one to ten classes. After consulting the fit indices and prior to selecting the optimal class model, we considered whether the classes were distinct and substantively meaningful. According to the BIC criterion, the seven-class model had the best relative fit, whereas the AIC did not reach a minimum level. The seven-class model was chosen. This choice was underpinned by the fact that the model can be given a meaningful interpretation based, among others, on experience from clinical practice in occupational medicine.

**Table 2:** Fit statistics and diagnostic criteria for latent class analyses.

Number of latent classes	Number of parameters estimated	LL	BIC	AIC	max(BVR)	Entropy	Class Error	DI	Smallest class size (%)
1	9	-97,490	195,072	194,997	4873.86	1.00	0.00	0.260	100
2	19	-90,751	181,698	181,540	1315.62	0.64	0.07	0.099	22
3	29	-90,005	180,308	180,067	253.13	0.58	0.12	0.068	12
4	39	-89,624	179,650	179,327	68.70	0.55	0.16	0.057	6
5	49	-89,382	179,269	178,862	34.80	0.54	0.17	0.040	4
6	59	-89,267	179,141	178,652	11.25	0.60	0.16	0.032	3
7	69	-89,167	**179,044**	178,472	4.02	0.59	0.18	0.027	2
8	79	-89,132	179,077	178,422	2.03	0.57	0.19	0.025	1
9	89	-89,108	179,134	178,395	1.85	0.57	0.19	0.024	1
10	99	-89,086	179,193	178,371	1.05	0.53	0.22	0.022	0.3
11–15	Not well identified								

LL = log-likelihood; BIC = Bayesian information criterion; AIC = Akaike information criterion; max(BVR) = the largest bivariate residual in the pairwise cross-table of the nine indicators; DI = Dissimilarity index

The selected model has some shortcomings. While a classification error of 0.18, a dissimilarity index value of 0.027, and a minimum class size of 2% are considered acceptable, the model does not fully explain all interrelationships between variables, particularly between poor social support and own disease, as indicated by a bivariate residual of 4.02 (max(BVR)). Residual values above 3.84 are statistically significant at the 0.05 level. Although we could potentially have improved the fit of the model to the data by relaxing the assumption of conditional independence between these two variables, we opted not to do so due to a lack of theoretical justification. The second largest residual in the seven-class model was 3.08.

In addition, the ability of the model to allocate individuals to a specific class was moderate, as evidenced by its 0.59 entropy value. While values above 0.8 are considered satisfactory [59], this value suggests that the model has several borderline cases or observations that are difficult to categorize into a single class. Despite these shortcomings, we believe that the model yields valuable insights.

### Characteristics of latent classes

Class proportions and the estimated probabilities of having experienced any particular stressor within the past 12 months are shown in Table 3. Class 1 was characterized by low probabilities across all stressors, whereas the other six classes reported high or moderate probabilities in one or more domains. All seven classes were labelled according to the stressor profiles (Table 3). Of note, Class 4 had a less distinctive profile than the other classes with the highest class-specific probability of 0.46 (poor social support). In contrast, Classes 2–3 and 5–7 all had "signature variables" with probabilities ranging from 0.76 to 0.94, while class-specific probabilities in Class 1 were low, ranging from 0.01 to 0.09.

**Table 3:** Latent class results characterizing seven stressor profiles (class proportions and class-specific probabilities of stressors).

	Latent Class						
Class	1	2	3	4	5	6	7
Assigned label	Low Stressor Burden	Burdened by Financial, Work, and Housing Stressors	Burdened by Disease and Death Among Close Relatives	Burdened by Lack of Social Support and Strained Relationships	Burdened by Own Disease	Complex Stressor Burden Involving Financial, Work, and Housing Stressors	Complex Stressor Burden Involving Own Disease and Disease and Death Among Close Relatives
Class proportion	0.64	0.10	0.09	0.08	0.06	0.02	0.02
Item-response probabilities							
Financial circumstances	0.04	**0.80**	0.05	0.17	0.11	***0*.*92***	**0.59**
Housing conditions	0.02	0.42	0.03	0.12	0.02	***0*.*77***	0.41
Work situation	0.07	**0.57**	0.11	0.28	0.27	***0*.*77***	0.47
Relationship with partner	0.02	0.12	0.06	0.38	0.00	***0*.*56***	0.36
Relationship with family and friends	0.01	0.05	0.04	0.29	0.04	***0*.*65***	0.47
Disease	0.04	0.30	0.19	0.27	***0*.*94***	**0.54**	**0.76**
Disease among close relatives	0.02	0.15	**0.77**	0.21	0.12	0.29	***0*.*92***
Deaths among close relatives	0.03	0.07	0.34	0.10	0.07	0.10	***0*.*60***
Perceived social support	0.09	0.19	0.09	0.46	0.22	***0*.*56***	0.45
Number of reported perceived stressors (mean)	0.32	2.69	1.67	2.28	1.78	5.16	5.04

Item-response probabilities > 0.5 in bold to facilitate interpretation. With each item, the class with the highest response probability is in italic.

### Population characteristics of latent stressor classes

Table 4 shows the socio-demographic composition of the seven latent classes and the results of the multivariate, multinomial logistic regression analysis.

**Table 4:** Demographics and multinomial logistic regression results for covariates by latent class of stressors (intercept omitted from table).

	Latent Class														
Class	1		2		3		4		5		6		7		
Assigned label	Low Stressor Burden		Burdened by Financial, Work, and Housing Stressors		Burdened by Disease and Death Among Close Relatives		Burdened by Lack of Social Support and Strained Relation-ships		Burdened by Own Disease		Complex Stressor Burden Involving Financial, Work, and Housing Stressors		Complex Stressor Burden Involving Own Disease and Disease and Death Among Close Relatives		Wald test p-value
Characteristics	%	OR	%	OR	%	OR	%	OR	%	OR	%	OR	%	OR	
Mean age (SD), years		49,4		34,9		57,6		40,3		58,9		36,3		46,0	
Age, years															
16–24	14		26		6		21		0		20		11		
25–34	13		30		6		14		4		34		16		
35–44	14		21		10		25		11		20		13		
45–54	17		16		17		23		18		17		24		
55–64	16		6		20		11		29		8		23		
65–74	17		1		23		5		16		1		8		
75+	10		0		18		1		21		0		4		
Age (per 5-year increase)		1.0		**0.8**		**1.1**		**0,9**		**1.0**		**0.8**		**0.9**	p<0.001
Sex															
Male	53	1,0	47	1,0	37	1,0	49	1,0	36	1,0	50	1,0	37	1,0	p<0.001
Female	47	1,0	53	**1,3**	63	**1,9**	51	1,0	64	**1,7**	50	0,9	63	**1,4**	
Ethnic origin															
Danish	93	1,0	82	1,0	95	1,0	83	1,0	95	1,0	75	1,0	70	1,0	p<0.001
Other Western	4	1,0	8	**2,3**	3	1,0	6	**2,3**	1	**0,7**	6	**3,4**	8	**3,0**	
Non-Western	4	1,0	10	**1,8**	2	**0,7**	11	**2,4**	4	0,9	19	**4,2**	23	**6,3**	
Educational attainment															
High (15+ years)	15	1,0	12	1,0	16	1,0	14	1,0	29	1,0	25	1,0	26	1,0	p<0.001
Medium (11–15 years)	55	1,0	56	1,1	54	**0,9**	53	0,9	51	1,3	54	1,7	47	0,9	
Low (0–10 years)	30	1,0	32	**0,9**	30	**0,8**	33	1,1	20	**1,6**	21	**1,9**	28	**1,5**	
Work status															
Working	63	1,0	61	1,0	53	1,0	75	1,0	27	1,0	31	1,0	40	1,0	p<0.001
Non-working	37	1,0	39	**2,1**	47	**0,9**	25	**0,9**	73	**2,9**	69	**5,7**	60	**2,5**	
Living with spouse/cohabitant															
Single	31	1,0	48	1,0	26	1,0	48	1,0	38	1,0	56	1,0	41	1,0	p<0.001
Married/cohabitating	69	1,0	52	**0,8**	74	**1,3**	52	**0,4**	62	**0,8**	44	**0,6**	59	**0,6**	
Living with child(ren) aged 0–15 years															
No	73	1,0	61	1,0	81	1,0	56	1,0	89	1,0	64	1,0	74	1,0	p<0.001
Yes	27	1,0	39	**1,3**	19	**0,9**	44	**2,1**	11	**0,8**	36	**2,0**	26	**1,4**	

* = p<0,05. OR = odds ratio. Each odds ratio is adjusted for the remaining variables in the model.

In the regression model, several factors were found to influence the probability of belonging to the classes:

*Sex*: Females were more likely to belong to Classes 2, 3, 5, and 7 and less likely to belong to Class 6, compared with Class 1.

*Age*: Higher age increased the likelihood of belonging to Class 3 but decreased the likelihood of belonging to Classes 2, 6, and 7, compared with Class 1

*Educational attainment*: Low educational attainment increased the likelihood of belonging to Classes 5 and 6, and decreased the likelihood of belonging to Class 3, compared with Class 1.

*Marital status*: Being married or cohabitating increased the likelihood of belonging to Class 3 and decreased the probability of belonging to Classes 2, 5, 7, and particularly Class 4, compared with Class 1

*Ethnic origin*: Non-Danish ethnic origin increased the likelihood of belonging to Classes 2, 4, and particularly Classes 6 and 7, compared with Class 1.

*Employment status*: Having a non-working status increased the probability of belonging to Classes 2, 5, 7, and particularly Class 6, while decreasing the probability of belonging to Classes 3 and 4, compared with Class 1.

*Living with children*: Living with children aged 0–15 years increased the likelihood of belonging to Classes 2, 4, 6, and 7 and decreased the probability of belonging to Classes 3 and 5, compared with Class 1.

### The association between latent classes and perceived stress level

**Fig 1:**
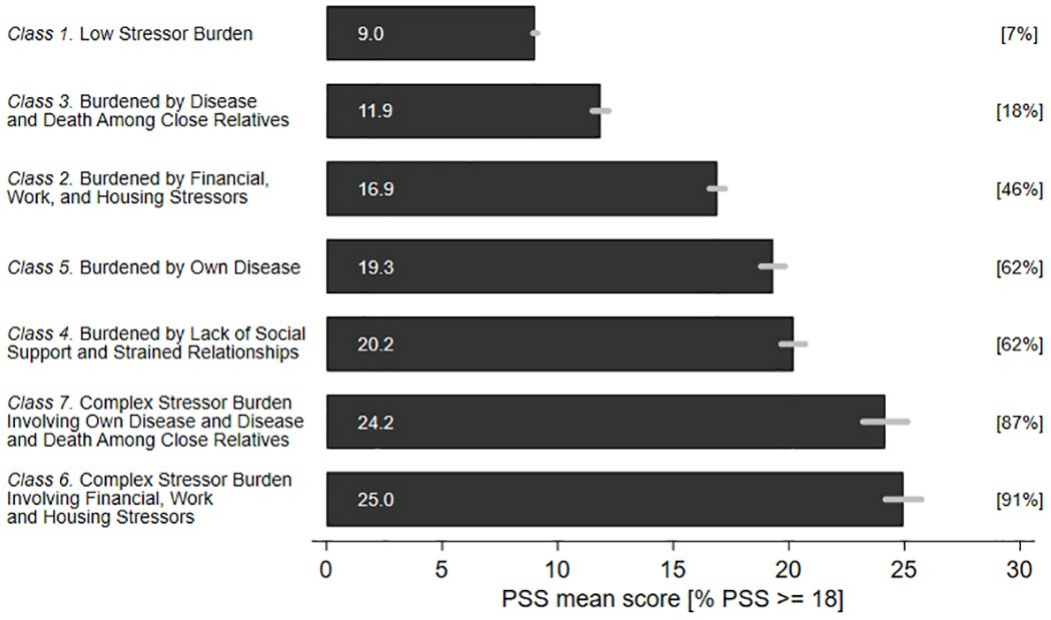
Perceived stress levels across latent classes.

The variation in perceived stress levels across latent classes is reported in Fig 1. A significant difference was observed in stress levels between the seven latent classes. For PSS; the average varied from 9.0 in Class 1 to 25.0 in Class 6. Correspondingly, the proportion with a high stress level varied from 7% in Class 1 to 91% in Class 6.

To assess the importance of the increased perceived stress level in Classes 2 to 7 compared with Class 1, we calculated Cohen’s d and utilized his rule of thumb, as expanded by Sawilowsky [60], for interpretation. The effect size of membership of Class 3 compared with Class 1 was slightly below medium (d = 0.41). Membership of Class 2 demonstrated a large effect size (d = 1.11), whereas membership of Class 4 and Class 5 showed very large effect sizes (d = 1.48; d = 1.44), and membership of Class 6 and Class 7 exhibited exceptionally large effect sizes (d = 2.35; d = 2.23).

## Discussion

This study used LCA to identify population segments with different stressor profiles. Seven latent classes were identified. These seven classes offer a detailed understanding of how various common sources of stress clustered across the general Danish population. Moreover, further analyses revealed marked differences in socio-economic composition and perceived stress levels across these classes. Altogether, this study presents novel insights that may be used to effectively address mental health concerns in the general population, focusing on groups in the population who find themselves in particularly challenging situations. In the following sections, we discuss the distinctive characteristics of each segment, ordered from lowest to highest perceived stress level. Furthermore, we apply a life-course perspective to the identified segments to enhance our understanding of the dynamic interaction between the segments.

### Stressor profiles in the population

*Class 1* comprised almost two-thirds of the population. In this segment, the stressor burden was low in all domains, and the segment had the lowest level of perceived stress of all seven classes. Compared with the entire adult population, the segment had a moderate over-representation of men, persons of Danish ethnic background, persons in work, and persons who were married/cohabiting.

*Class 3* comprised 9% of the population. In this segment, the stressor burden was very high for disease among close relatives and high for deaths among close relatives but low in the other domains. The perceived stress level was somewhat elevated compared with Class 1. The group was characterized by a predominance of women and older people. This group also had the largest proportion of married/cohabiting individuals. The group included people who cared for an ill partner, parent, child, friend, etc., or who mourned the loss of a loved one.

It is estimated that around one in six people of the adult population in the 28 EU member states are informal caregivers [61], which has been identified as a chronic stressor that places them at risk for physical and emotional problems [62, 63]. On this backdrop, it is noteworthy that only one in five citizens in Class 3 had a high level of perceived stress. Thus, the majority of Class 3 appeared to cope well with their caregiver burden or the worries and grief associated with illness and death of a loved one. Moreover, they had managed to balance this with other commitments, perhaps because they did not experience other major stressors in their lives. It is, however, important not to trivialize the caregiver burden. In combination with other stressors, it can pose a serious threat to mental well-being. Older people may generally be better than younger people at navigating around stressors in their lives [64–66]. However, many older people have fewer resources than younger people to withstand adversity and may therefore be more easily "knocked over" by unforeseen stressors [67]. Older individuals who are burdened by caregiving are therefore less robust if their circumstances change for the worse, e.g., if they are affected by illness. Moreover, grief in adults is associated with depression and other psychological disorders [68, 69].

Points of attention: An increasing number of people require or will require long-term care. Society is heavily dependent on informal caregivers and should recognize and support their efforts. Half of Class 3 had a job, and for this group, it is important to focus on improving work-life balance. It is important to acknowledge the grief of those who have lost a loved one and provide psychological treatment for those experiencing complicated grief reactions.

*Class 2* included 10% of the population. The stressor burden was very high for finances, work, and housing. The perceived stress level was significantly elevated compared with Class 1. Class 2 was the youngest of the seven classes as eight out of ten people were 16–44 years old. Women, people residing with dependent children, and people who were single were moderately overrepresented, whereas people of a non-Danish background appeared twice as often as in the general population. Class 2 included young people who were in education or at the beginning of their professional careers and who were struggling with the challenges of doing well in the educational system, establishing themselves on the labor market, finding a partner, becoming parents, and obtaining affordable and satisfactory housing. Most young people experience a high degree of well-being, but the transition into adulthood can be challenging for some [70].

Researchers have pointed out that the transition into adulthood has become much more protracted in recent decades, and that it is now often delayed until the late twenties or early thirties [71]. Additionally, the more or less standardized passages into adulthood of earlier times have been replaced by a more diversified and highly individualized ’in-between’ period, referred to as emerging adulthood by some researchers [72, 73]. Finding employment remains an important condition upon which other transitions into adulthood build [71], but changes in the labor market have increased the number of insecure and ’precarious’ jobs, which disproportionately affect young people [74]. Several longitudinal studies indicated that entry into a labor market with precarious employment is associated with an increased risk of subsequent mental health problems [75–77].

Points of attention: A group of predominantly young people are struggling to create a stable financial basis for their existence. It is important to maintain a political focus on conditions that can ensure young people a safe transition into adulthood, including access to education, a labor market with secure jobs, and affordable housing.

*Class 5* comprised 6% of the population. Almost everyone in this class reported being burdened by disease. The perceived stress level was much greater than in Class 1. Class 5 corresponded in composition to Class 3 with a predominance of women, older, and married/cohabiting individuals. However, the group had a lower level of education and a higher percentage of non-working people (three out of four did not work).

Most diseases may cause a degree of uncertainty, disruption of life routines, and lack of control, at least for a period of time [78, 79]. Chronic disease, in particular, requires changes across psychological, social, and physical aspects of a person’s life [80]. Therefore, living with one or more chronic conditions is often perceived as stressful. This may be due to functional limitations and symptoms related to the disease itself (disease burden). However, a growing body of research also indicates that treatment of the disease often places a burden on the patient as it includes attending appointments, undergoing medical investigations and procedures, self-monitoring, adopting lifestyle advice, navigating healthcare systems, taking medications, and enduring any adverse effects [81–83]. In addition, chronic disease frequently results in financial burdens (although this is less common in Class 5 than in Class 7 where disease coincides with multiple other stressors), presents existential challenges, and entails fundamental changes in self-perception (e.g. described as ‘biographical disruption’) [84].

Points of attention: An increasing number of people are diagnosed and treated for chronic conditions [85] and chronic disease is a major source of stress [9, 10]. It is important that healthcare remains aware of patients’ psychological well-being. Access to mental support is required for patients who need it. It is also important to minimize the treatment burden, address patients’ priorities, support self-management, and underpin healthy behaviors. Furthermore, it is essential to implement policy measures aimed at enhancing employment opportunities for individuals with health challenges. Employers must also acknowledge that illness can be a significant source of stress among their employees.

*Class 4* included 8% of the population with a stressor burden mainly characterized by lack of social support and strained relationships. The perceived stress level was very high compared with Class 1. Class 4 was the second youngest of the seven classes. It spanned people from young adulthood to middle-aged people, with an equal distribution of men and women. Persons of non-Danish background appeared almost twice as often as in Class 1. Class 4 had the highest prevalence of people living with a dependent child.

As mentioned above, Class 4 had a less marked profile than the other classes with the highest class-specific probability of 0.46 (poor social support). Therefore, caution is needed when interpreting these findings. Even so, Class 4 seems to include people burdened by social relations–relationship with partner, relationship with family and friends, and poor social support. Forming and maintaining close relationships–with parents, siblings, friends, partners, children, etc.–is an important part of life for most people [86] and a source of joy and satisfaction but also a source of social negativity (including frustration, conflict, pain, and suffering) and grief. A review of stress research in couples found that conflicts and tensions often arose between the partners from expressed differences in goals, attitudes, needs, desires, habits, and personality traits of one’s partner. Conflicts also arose due to insufficient support for household tasks, stress coping, or compatibility issues among partners [87].

Transitions in close relationships are a well-known source of stress [88]. For instance, the transition into parenthood is among the most challenging experiences for committed partners [89, 90]. The changing of close relationships from harmonious to conflict-ridden may result in the loss of a crucial source of social support, impacting mental well-being negatively, while the end of close relationships may lead to separation distress. A recent research overview of the psychology of close relationships emphasized the importance of studying relationships within their contexts (e.g., relationship type, life stage, and culture) [86].

Points of attention: At any point in time, a certain part of the population lives in strained relationships with their partner, parents, or another primary person; or suffers the consequences of social disconnectedness (e.g. lack of social support). Supportive environments can help people through periods of relational difficulties, and the promotion of social inclusivity, trust, and interconnectedness within various settings, such as residential areas, educational institutions, and workplaces, is important for enhancing well-being and social health. For some individuals, seeking professional assistance, such as counseling or psychotherapy, has proved highly beneficial. Ensuring equal and convenient access to these services for those in need is paramount for fostering a supportive and healthy society.

*Class 7* comprised 2% of the population. This was the first of two classes with a complex stressor burden. This class was characterized by a particularly high burden of own disease, disease among and death of close relatives, and financial burdens. The perceived stress level was exceptionally high compared with Class 1. Class 7 corresponded in composition to Class 5 (Burdened by Own Disease) and partly to Class 3 (Burdened by Disease and Death among Close Relatives) with a majority of women, married/cohabiting persons, and a high proportion with low education. The age composition, however, was significantly younger with an age profile dominated by middle-aged and young people. Furthermore, Class 7 had a significantly higher proportion of people of non-Danish ethnic origin and people with children in the household. This indicates that Class 7 was not just an amalgamation of Class 3 and 5—"double-hit people"—but a group that has followed a different developmental path.

Points of attention: This class consists of people in a very vulnerable situation with an increased risk of developing–or who have already developed–pathological stress reactions. Prevention and treatment strategies have recently been suggested for people who experience multiple stressors in regular, daily life [91]. These strategies include keeping close relationships active, public and media educational programs aimed at enhancing individual resilience by utilization of social networks, and, if treatment is needed, psychotherapy in the form of interpersonal psychotherapy and cognitive behavioral therapy.

Based on the stressor profile, a public health strategy aimed at reducing the stressor burden in Class 7 should prioritize initiatives within the healthcare system. These efforts should address social support gaps and mental health challenges related to disease management and treatment, as well as the unique stressors associated with being a relative or caregiver. Such initiatives must also take into account the high proportion of individuals in this group who have relatively limited resources and face complex stressors, including financial burdens.

Evidence from a meta-analysis [92] highlights the short-term benefits of mental health interventions for informal caregivers, such as preventive psychology, recovery-focused therapy, and psychoeducation. However, the authors also stress the need to weigh potential adverse effects against these benefits, noting the absence of a gold standard for improving mental health outcomes in informal caregivers.

Given this context, we believe it is crucial to provide targeted support for both patients and their relatives in managing the mental health challenges that arise during and after interactions with the healthcare system. Such support could enhance their overall resilience and reduce the cumulative stressor burden experienced by this vulnerable group.

*Class 6* included 2% of the population. This was the second of two classes with a complex stressor burden. This group was characterized by a particularly high burden due to financial circumstances, housing conditions, work situation, strained relations with their partner, family, and friends, but also of own disease and poor social support. The perceived stress level was exceptionally high compared with Class 1.

Class 6 was dominated by young people with a particularly large overrepresentation of individuals aged 25–34 years. Individuals in this class faced personal adversity on many fronts and seemed to have embarked on a path of numerous hardships early in life. According to a recent research overview, the social context of young people’s mental health has far-reaching consequences [93]. Findings link peer and parenting problems in youth to adult mental health problems [94]. A cohort study [95] showed that young people who face social adversities, such as parental illness, unemployment, and housing shortages, tend to develop mental health problems. Studies of young adults emphasized the importance of supportive relationships with family and friends [96]. While stable, supportive peer relationships provided protection, family and peer problems raised stress levels [97, 98].

Points of attention: A small group of predominantly young people is on a risky and difficult developmental path and needs help to cope with the challenges in their lives. Similar to Class 7, this group requires a dedicated and comprehensive preventive and therapeutic intervention from society.

Based on the stressor profile, a public health strategy aimed at reducing the stressor burden in Class 6 should adopt a dual approach. One branch should focus on children and young people in vulnerable situations, while the other should target predominantly younger adults on the margins of the labor market and in precarious circumstances.

For children and young people, initiatives should emphasize family-centered interventions and be integrated within day care services, schools, and community-based social programs. These efforts could provide a foundational support system for addressing stressors early in life. For younger adults, initiatives should combine social support with labor market interventions, aiming to reduce the compounded stressors associated with unemployment and economic instability.

Recent evidence from an umbrella review of community-based interventions [99] highlights the strongest outcomes for initiatives addressing financial insecurity and providing welfare support, both of which were shown to improve mental health outcomes. Incorporating such evidence-based strategies into targeted programs could enhance their efficacy in alleviating stressor burdens for these vulnerable groups.

### A life-course perspective on stressor profiles

**Fig 2:**
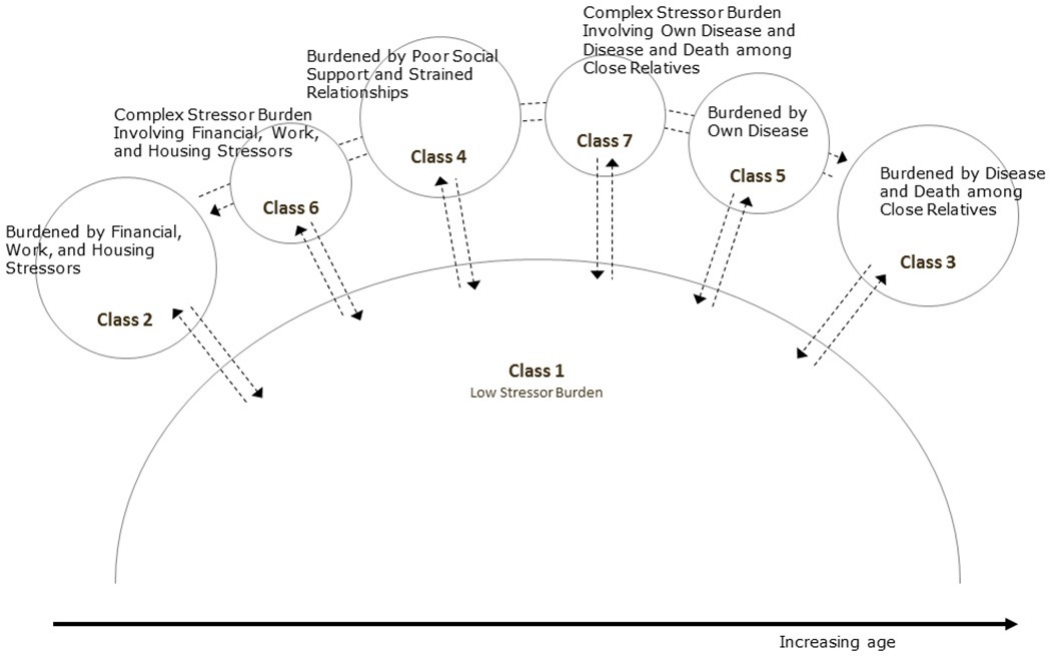
A life-course perspective on stressor profiles.

By adopting a life-course perspective, the results of the present study may be interpreted dynamically, cf. Fig 2. From infancy to old age, individuals adapt to various challenges and stressors at different life stages [100]. Developmental research on mental health points to the heterogeneity in the developmental processes of the life course [101]. The specific stressor patterns identified in this study likely reflect both this uniformity and variation. Most of the time, most people are able to stay within their mental comfort zone as they navigate and negotiate their way through life (Class 1), whereas others, influenced by personal choices and external factors, will be pushed to their limits or beyond for shorter or longer periods, some even permanently (Class 2–7). While Class 1 included all age groups, Classes 2 and 6 were dominated by young people, Class 4 was dominated by young and middle-aged people, Class 7 by middle-aged people, and Classes 3 and 5 were dominated by older people. It may be hypothesized that individuals transition into and out of the segments as they move along their life course with Class 1 as a kind of default state. This hypothesis is supported by the fact that we previously found that within-person changes in stressor burden were considerable over a four-year period [10]. Furthermore, the hypothesis is in line with the theoretical framework of our study since the ability to cope with stressors will most likely change during the life course and is thus related to social characteristics and life circumstances at any given time. In this perspective, the life-course approach underlines the importance of not focusing exclusively on single stress factors, such as occupational stress, in stress mitigation policies and interventions. Instead, it is crucial to adopt a comprehensive approach consistent with the previously mentioned theories; one that underscores the combined effects of multiple stressors and recognizes the importance of life-course context. It is critical to ensure that individuals do not remain stuck in a high-stress segment, causing chronic stress, or to prevent them from transitioning into another high-stress segment. This dynamic interpretation of stressor profiles should be tested in future longitudinal research.

To our knowledge, this is the first study to analyze how sources of stress cluster and associate with perceived stress in a general population using LCA. A strength of our study is that it included a large sample size (32,417 individuals) and a wide age range from a representative sample of the general population.

However, our study also carries some limitations. The included stressors are self-reported, which implies potential response bias. The analyses were also limited by the number of stressors included. Furthermore, the LCA model adopted does not fully explain all relationships between stressors. Finally, the model has low class separation, which means that it poorly predicts individuals’ class affiliation and therefore has many borderline cases. To enhance the robustness the presented model, replication studies using additional data sets would be beneficial. Specifically, incorporating a broader range of perceived stressors and more diverse variables related to social support–such as material, emotional and practical support across various context like work and family–would be particularly valuable.

Theoretically, greater stressor diversity is associated with better affective well-being compared to an equivalent number of stressors concentrated within a single domain, as proposed by Koffer et al. [13]. However, our findings reveal deviations from this theoretical framework. Specifically, some classes exhibit low perceived stress levels despite low stressor diversity (e.g., Class 3), while others report high perceived stress levels despite high stressor diversity (e.g., Class 4).

Class 3, characterized by disease and deaths among relatives as the predominant stressors, reports lower perceived stress levels compared to all other classes except Class 1. In contrast, Class 4, which exhibits moderate probabilities across multiple stress domains—including higher probabilities for poor social support—reports elevated perceived stress levels.

These findings suggest that certain stressors may disproportionately influence perceived stress levels, overriding the potential benefits of diversity. Prior research [9] indicates that stressors such as personal illness, perceived social support, and work situation are the most significant contributors to perceived stress. In comparison, stressors such as disease and deaths among relatives account for a smaller proportion of the variance in perceived stress levels.

Our results underscore the importance of refining the stressor diversity theory by accounting for the differential impact of individual stressors on perceived stress. This nuance could enhance the explanatory power of the theory and provide a more comprehensive understanding of the relationship between stressor diversity and well-being.

## Conclusion

Utilizing latent class analysis, we identified seven distinct population segments characterized by different stressor profiles. Six of the segments exhibited moderately to highly elevated perceived stress levels but differed in terms of socioeconomic composition and stressor profiles. Of note, 4% of the population grappled with a complex interplay of stressors and an exceptionally high level of perceived stress. The segments identified may be hypothesized to present a snapshot of a dynamic pattern in which individuals transition in and out of segments with differing stressor characteristics as they move along their life course. These findings may inform strategies aimed at enhancing overall mental health in the general population by addressing issues and difficulties that are unique to specific population segments facing particularly challenging life situations.

## Supporting information

S1 File Data availability. (DOCX)

## References

[1] Bhugra D, Bhui K, Wong SYS, Gilman SE. Oxford Textbook of Public Mental Health: OUP Oxford; 2018.

[2] Santosa A, Rosengren A, Ramasundarahettige C, Rangarajan S, Chifamba J, Lear SA, et al. Psychosocial Risk Factors and Cardiovascular Disease and Death in a Population-Based Cohort From 21 Low-, Middle-, and High-Income Countries. Jama Network Open. 2021;4(12). 10.1001/jamanetworkopen.2021.38920 PMC8674745 34910150

[3] Weitzel EC, Lobner M, Glaesmer H, Hinz A, Zeynalova S, Henger S, et al. The Association of Resilience with Mental Health in a Large Population-Based Sample (LIFE-Adult-Study). International Journal of Environmental Research and Public Health. 2022;19(23). 10.3390/ijerph192315944 PMC9740913 36498017

[4] Honnudóttir V, Veyhe AS, Mohr M, Strøm M, Mohr M. Effects of the COVID-19-induced lockdown period on general well-being, perceived stress and activity levels in the Faroe Islands. Scandinavian Journal of Public Health. 2023;51(5):648–55. doi: 10.1177/14034948231176708 37264919 PMC10240299

[5] Jensen HAR, Davidsen M, Møller SR, Román JEI, Kragelund K, Christensen AI, et al. Danskernes sundhed–Den Nationale Sundhedsprofl 2021. København: Sundhedsstyrelsen; 2022.

[6] Stress in America^™^ 2020: A National Mental Health Crisis. Washington, DC: American Psychological Association; 2020.

[7] WHO European framework for action on mental health 2021–2025. Copenhagen: WHO Regional Office for Europe; 2022.

[8] Carr D, Umberson D. The social psychology of stress, health, and coping. Handbook of social psychology, 2nd ed. Handbooks of sociology and social research. New York, NY, US: Springer Science + Business Media; 2013. p. 465–87.

[9] Sørensen JB, Lasgaard M, Willert MV, Larsen FB. The relative importance of work-related and non-work-related stressors and perceived social support on global perceived stress in a cross-sectional population-based sample. BMC public health. 2021;21(1):1–14. 33740936 10.1186/s12889-021-10594-2 PMC7980655

[10] Larsen FB, Lasgaard M, Willert MV, Sørensen JB. Estimating the causal effects of work-related and non-work-related stressors on perceived stress level: A fixed effects approach using population-based panel data. PLOS ONE. 2023;18(8):e0290410. doi: 10.1371/journal.pone.0290410 37616304 PMC10449195

[11] Lazarus RS, Folkman S. Stress, Appraisal, and Coping. New York, NY: Springer Publishing Company; 1984.

[12] Pearlin LI. The Sociological Study of Stress. J Health Soc Behav. 1989;30(3):241–56. 2674272

[13] Koffer RE, Ram N, Conroy DE, Pincus AL, Almeida DM. Stressor Diversity: Introduction and Empirical Integration Into the Daily Stress Model. Psychol Aging. 2016;31(4):301–20. doi: 10.1037/pag0000095 27294713 PMC4943459

[14] Brose A, Scheibe S, Schmiedek F. Life Contexts Make a Difference: Emotional Stability in Younger and Older Adults. Psychol Aging. 2013;28(1):148–59. doi: 10.1037/a0030047 23066802

[15] Wrzus C, Muller V, Wagner GG, Lindenberger U, Riediger M. Affective and cardiovascular responses to daily hassles: complexity of event matters for age effects. Gerontologist. 2011;51:517-.

[16] Gove WR. Gender differences in mental and physical illness—the effects of fixed roles and nurturant roles. Social Science & Medicine. 1984;19(2):77–91. doi: 10.1016/0277-9536(84)90273-9 6474235

[17] Bartley M, Popay J, Plewis I. Domestic conditions, paid employment and womens experience of ill-health. Sociology of Health & Illness. 1992;14(3):313–43.

[18] Pearlin LI, Aneshensel CS, Leblanc AJ. The forms and mechanisms of stress proliferation: The case of AIDS caregivers. Journal of Health and Social Behavior. 1997;38(3):223–36. 9343962

[19] von Eye A, Bogat GA. Person-oriented and variable-oriented research: Concepts, results, and development. Merrill-Palmer Quarterly-Journal of Developmental Psychology. 2006;52(3):390–420.

[20] Weller BE, Bowen NK, Faubert SJ. Latent Class Analysis: A Guide to Best Practice. Journal of Black Psychology. 2020;46(4):287–311.

[21] Prandy K. Ideal types, stereotypes and classes. British Journal of Sociology. 2002;53(4):583–601. doi: 10.1080/0007131022000021498 12556284

[22] Vanroelen C, Louckx F, Moors G, Levecque K. The clustering of health-related occupational stressors among contemporary wage-earners. European Journal of Work and Organizational Psychology. 2010;19(6):654–74.

[23] Jenull BB, Wiedermann W. The Different Facets of Work Stress: A Latent Profile Analysis of Nurses’ Work Demands. Journal of Applied Gerontology. 2015;34(7):823–43. doi: 10.1177/0733464813495472 24652894

[24] Mayerl H, Stolz E, Waxenegger A, Freidl W. Exploring differential health effects of work stress: a latent class cluster approach. Peerj. 2017;5. doi: 10.7717/peerj.3106 28344906 PMC5363255

[25] Collie JR, Mans FC. Teacher and school stress profiles: A multilevel examination and associations with work-related outcomes. Teaching and Teacher Education. 2022;116.

[26] Shahidi FV, Gignac MAM, Oudyk J, Smith PM. Assessing the Psychosocial Work Environment in Relation to Mental Health: A Comprehensive Approach. Annals of Work Exposures and Health. 2021;65(4):418–31. doi: 10.1093/annweh/wxaa130 33555321 PMC8091465

[27] Pajovic V, Shuey KM. Patterns and stratification of stressor exposure among Canadian workers. Canadian Review of Sociology-Revue Canadienne De Sociologie. 2021;58(1):86–104. doi: 10.1111/cars.12323 33600082

[28] Marzocchi I, Ghezzi V, Di Tecco C, Ronchetti M, Ciampa V, Olivo I, et al. Demand-Resource Profiles and Job Satisfaction in the Healthcare Sector: A Person-Centered Examination Using Bayesian Informative Hypothesis Testing. International Journal of Environmental Research and Public Health. 2023;20(2). doi: 10.3390/ijerph20020967 36673725 PMC9858661

[29] Pulido-Martos M, Cortes-Denia D, Luque-Reca O, Lopez-Zafra E. Authentic leadership and personal and job demands/resources: A person-centered approach and links with work-related subjective well-being. Current Psychology.

[30] Wenger M, Sauli F, Berger JL. Apprentice profiles reflecting role stress in dual vocational education and training in Switzerland. Psychologie Du Travail Et Des Organisations. 2022;28(1):73–86.

[31] Shan YW, Shang J, Yan Y, Lu GD, Hu DY, Ye XC. Mental workload of frontline nurses aiding in the COVID-19 pandemic: A latent profile analysis. Journal of Advanced Nursing. 2021;77(5):2374–85. doi: 10.1111/jan.14769 33594687 PMC8014576

[32] Liao PC, Chen SK, Lin SSJ. Latent profiles of stress and their relationships with depression and problematic Internet use among college freshmen. Scandinavian Journal of Psychology. 2018;59(6):621–30. 30252933 10.1111/sjop.12489

[33] Brown LL, Garcia C, Reeves AN, Pamplin JR, Mitchell UA. Stress is a Latent Construct: Exploring the Differential Experience of Stress and Discrimination on Depressive Symptoms Among Black Older Adults. Journal of Aging and Health. 2022;34(3):334–46. doi: 10.1177/08982643221086333 35418259 PMC9379938

[34] Ruijter E, Mulder EA, Vermunt JK, van Nieuwenhuizen C. Many, more, most: four risk profiles of adolescents in residential care with major psychiatric problems. Child and Adolescent Psychiatry and Mental Health. 2017;11. 10.1186/s13034-017-0204-1 PMC5738157 29270217

[35] Christensen AI, Lau CJ, Kristensen PL, Johnsen SB, Wingstrand A, Friis K, et al. The Danish National Health Survey: Study design, response rate and respondent characteristics in 2010, 2013 and 2017. Scand J Pub Health. 2020:1403494820966534. doi: 10.1177/1403494820966534 33161874

[36] Bekendtgørelse af lov om videnskabsetisk behandling af sundhedsvidenskabelige forskningsprojekter og sundhedsdatavidenskabelige forskningsprojekter [Proclamation of the Act on scientific ethical treatment of health science research projects and health data science research projects]: Retsinformation; 2020 [https://www.retsinformation.dk/eli/lta/2020/1338#P14.

[37] Pedersen CB. The Danish Civil Registration System. Scand J Public Health. 2011;39(7 Suppl):22–5. doi: 10.1177/1403494810387965 21775345

[38] Särndal C-E, Lundström S. Estimation in surveys with nonresponse: John Wiley & Sons; 2005.

[39] Norbeck JS. Modification of life event questionnaires for use with female respondents. Res Nurs Health. 1984;7(1):61–71. doi: 10.1002/nur.4770070110 6565302

[40] Sondergaard C, Olsen J, Friis-Hasche E, Dirdal M, Thrane N, Sorensen HT. Psychosocial distress during pregnancy and the risk of infantile colic: a follow-up study. Acta Paediatr. 2003;92(7):811–6. doi: 10.1080/08035250310003857 12892160

[41] Tegethoff M, Greene N, Olsen J, Meyer AH, Meinlschmidt G. Maternal psychosocial stress during pregnancy and placenta weight: evidence from a national cohort study. PloS one. 2010;5(12):e14478–e. doi: 10.1371/journal.pone.0014478 21217829 PMC3013108

[42] Tegethoff M, Greene N, Olsen J, Schaffner E, Meinlschmidt G. Stress during pregnancy and offspring pediatric disease: A National Cohort Study. Environ Health Perspect. 2011;119(11):1647–52. doi: 10.1289/ehp.1003253 21775267 PMC3226491

[43] Eurostat. International Standard Classification of Education (ISCED) 2018 [updated 28-08-2018. https://ec.europa.eu/eurostat/statistics-explained/index.php/International_Standard_Classification_of_Education_(ISCED)).

[44] Cohen S, Kamarck T, Mermelstein R. A global measure of perceived stress. J Health Soc Behav. 1983;24(4):385–96. 6668417

[45] Lazarus RS. Psychological stress and the coping process. New York, NY, US: McGraw-Hill; 1966.

[46] Lazarus RS. Psychological stress and coping in adaption and illness. In: Lipowski ZJ, Lipsitt DR, Whybrow PC, editors. Psychosomatic medicine: current trends and clinical applications. New York: Oxford Univ. Press; 1977. p. 14–26.

[47] Maehlisen MH, Pasgaard AA, Mortensen RN, Vardinghus-Nielsen H, Torp-Pedersen C, Boggild H. Perceived stress as a risk factor of unemployment: a register-based cohort study. BMC Pub Health. 2018;18(1):728. doi: 10.1186/s12889-018-5618-z 29895286 PMC5998595

[48] Prior A, Fenger-Grøn M, Larsen KK, Larsen FB, Robinson KM, Nielsen MG, et al. The Association Between Perceived Stress and Mortality Among People With Multimorbidity: A Prospective Population-Based Cohort Study. Am J Epidemiol. 2016;184(3):199–210. doi: 10.1093/aje/kwv324 27407085

[49] Pedersen AB, Baggesen LM, Ehrenstein V, Pedersen L, Lasgaard M, Mikkelsen EM. Perceived stress and risk of any osteoporotic fracture. Osteoporos Int. 2016;27(6):2035–45. doi: 10.1007/s00198-016-3490-1 26786258

[50] Christiansen J, Larsen FB, Lasgaard M. Do stress, health behavior, and sleep mediate the association between loneliness and adverse health conditions among older people? Soc Sci Med. 2016;152:80–6. doi: 10.1016/j.socscimed.2016.01.020 26849687

[51] Lee EH. Review of the psychometric evidence of the perceived stress scale. Asian Nurs Res (Korean Soc Nurs Sci). 2012;6(4):121–7. doi: 10.1016/j.anr.2012.08.004 25031113

[52] Nielsen MG, Ornbol E, Vestergaard M, Bech P, Larsen FB, Lasgaard M, et al. The construct validity of the Perceived Stress Scale. J Psychosom Res. 2016;84:22–30. doi: 10.1016/j.jpsychores.2016.03.009 27095155

[53] Sinha P, Calfee CS, Delucchi KL. Practitioner’s Guide to Latent Class Analysis: Methodological Considerations and Common Pitfalls. Crit Care Med. 2021;49(1):e63–e79. doi: 10.1097/CCM.0000000000004710 33165028 PMC7746621

[54] Nylund KL, Asparouhov T, Muthén BO. Deciding on the Number of Classes in Latent Class Analysis and Growth Mixture Modeling: A Monte Carlo Simulation Study. Structural Equation Modeling: A Multidisciplinary Journal. 2007;14(4):535–69.

[55] Vermunt JK. Latent Class Modeling with Covariates: Two Improved Three-Step Approaches. Political Analysis. 2017;18(4):450–69.

[56] Bolck A, Croon M, Hagenaars J. Estimating Latent Structure Models with Categorical Variables: One-Step Versus Three-Step Estimators. Political Analysis. 2004;12(1):3–27.

[57] Bakk Z, Oberski DL, Vermunt JK. Relating Latent Class Assignments to External Variables: Standard Errors for Correct Inference. Political Analysis. 2014;22(4):520–40.

[58] Bakk Z, Kuha J. Relating latent class membership to external variables: An overview. Br J Math Stat Psychol. 2021;74(2):340–62. doi: 10.1111/bmsp.12227 33200411 PMC8247311

[59] Aflaki K, Vigod S, Ray JG. Part II: a step-by-step guide to latent class analysis. Journal of Clinical Epidemiology. 2022;148:170–3. doi: 10.1016/j.jclinepi.2022.05.009 35662622

[60] Sawilowsky SS. New Effect Size Rules of Thumb. Journal of Modern Applied Statistical Methods. 2009;8(2):597–9.

[61] Zigante V. Informal care in Europe: Exploring formalisation, availability and quality. 2018.

[62] Allen AP, Curran EA, Duggan Á, Cryan JF, Chorcoráin AN, Dinan TG, et al. A systematic review of the psychobiological burden of informal caregiving for patients with dementia: Focus on cognitive and biological markers of chronic stress. Neuroscience & Biobehavioral Reviews. 2017;73:123–64. 27986469 10.1016/j.neubiorev.2016.12.006

[63] Pinquart M, Sörensen S. Gender Differences in Caregiver Stressors, Social Resources, and Health: An Updated Meta-Analysis. The Journals of Gerontology: Series B. 2006;61(1):P33–P45. doi: 10.1093/geronb/61.1.p33 16399940

[64] Almeida DM, Horn MC. Is daily life more stressful during middle adulthood? In: Brim OG, Ryff CD, Kessler RC, editors. How healthy are we?: A national study of well-being at midlife. Chicago: The University of Chicago Press; 2004. p. 425–51.

[65] Lazarus RS. The role of coping in the emotions and how coping changes over the life course. In: Magai C, McFadden SH, editors. Handbook of emotion, adult development, and aging. San Diego: Academic Press; 1996. p. 289–306.

[66] Birditt KS, Fingerman KL, Almeida DM. Age differences in exposure and reactions to interpersonal tensions: a daily diary study. Psychol Aging. 2005;20(2):330–40. doi: 10.1037/0882-7974.20.2.330 16029096

[67] Charles ST. Strength and vulnerability integration: a model of emotional well-being across adulthood. Psychol Bull. 2010;136(6):1068–91. doi: 10.1037/a0021232 21038939 PMC3059514

[68] Komischke-Konnerup KB, Zachariae R, Johannsen M, Nielsen LD, O’Connor M. Co-occurrence of prolonged grief symptoms and symptoms of depression, anxiety, and posttraumatic stress in bereaved adults: A systematic review and meta-analysis. Journal of Affective Disorders Reports. 2021;4:100140.

[69] Schladitz K, Löbner M, Stein J, Weyerer S, Werle J, Wagner M, et al. Grief and loss in old age: Exploration of the association between grief and depression. J Affect Disord. 2021;283:285–92. doi: 10.1016/j.jad.2021.02.008 33578340

[70] Goossens L. Transition to Adulthood: Developmental Factors. In: Corijn M, Klijzing E, editors. Transitions to Adulthood in Europe. Dordrecht: Springer Netherlands; 2001. p. 27–42.

[71] Furlong A. Routledge Handbook of Youth and Young Adulthood. London: Routledge; 2017.

[72] Corijn M. Transition to Adulthood: Sociodemographic Factors. In: Corijn M, Klijzing E, editors. Transitions to Adulthood in Europe. Dordrecht: Springer Netherlands; 2001. p. 1–25.

[73] Arnett JJS, Moin. Emerging Adulthood: Oxford University Press; 2015.

[74] Standing G. Understanding the Precariat through Labour and Work. Development and Change. 2014;45(5):963–80.

[75] Thern E, Matilla-Santander N, Hernando-Rodriguez JC, Almroth M, Bodin T. Precarious employment in early adulthood and later mental health problems: a register-linked cohort study. Journal of Epidemiology and Community Health. 2023:jech-2023-220817. doi: 10.1136/jech-2023-220817 37567755 PMC10646894

[76] Canivet C, Bodin T, Emmelin M, Toivanen S, Moghaddassi M, Östergren P-O. Precarious employment is a risk factor for poor mental health in young individuals in Sweden: a cohort study with multiple follow-ups. BMC Public Health. 2016;16(1):687. 27485322 10.1186/s12889-016-3358-5 PMC4969669

[77] Dalglish SL, Melchior M, Younes N, Surkan PJ. Work characteristics and suicidal ideation in young adults in France. Social Psychiatry and Psychiatric Epidemiology. 2015;50(4):613–20. doi: 10.1007/s00127-014-0969-y 25308058

[78] Stress Vilchinsky N. and Management Crisis. In: Llewellyn CD, McManus C, Weinman J, Petrie KJ, Newman S, Ayers S, et al., editors. Cambridge Handbook of Psychology, Health and Medicine. Cambridge Handbooks in Psychology. 3 ed. Cambridge: Cambridge University Press; 2019. p. 309–12.

[79] Petrie KJ, Jones ASK. Coping With Chronic Illness. In: Llewellyn CD, McManus C, Weinman J, Petrie KJ, Newman S, Ayers S, et al., editors. Cambridge Handbook of Psychology, Health and Medicine. Cambridge Handbooks in Psychology. 3 ed. Cambridge: Cambridge University Press; 2019. p. 110–3.

[80] Whittemore R, Dixon J. Chronic illness: the process of integration. J Clin Nurs. 2008;17(7b):177–87. doi: 10.1111/j.1365-2702.2007.02244.x 18578794 PMC3648221

[81] May CR, Eton DT, Boehmer K, Gallacher K, Hunt K, MacDonald S, et al. Rethinking the patient: using Burden of Treatment Theory to understand the changing dynamics of illness. BMC Health Services Research. 2014;14(1):281. 24969758 10.1186/1472-6963-14-281 PMC4080515

[82] Kate SHM, Susannah CR, Simon DSF. Influence of health-system change on treatment burden: a systematic review. British Journal of General Practice. 2023;73(726):e59. 10.3399/BJGP.2022.0066 PMC9591018 36253115

[83] Sav A, King MA, Whitty JA, Kendall E, McMillan SS, Kelly F, et al. Burden of treatment for chronic illness: a concept analysis and review of the literature. Health Expectations. 2015;18(3):312–24. doi: 10.1111/hex.12046 23363080 PMC5060781

[84] Bury M. Chronic illness as biographical disruption. Sociol Health Illn. 1982;4(2):167–82. doi: 10.1111/1467-9566.ep11339939 10260456

[85] James SL, Abate D, Abate KH, Abay SM, Abbafati C, Abbasi N, et al. Global, regional, and national incidence, prevalence, and years lived with disability for 354 diseases and injuries for 195 countries and territories, 1990–2017: a systematic analysis for the Global Burden of Disease Study 2017. Lancet. 2018;392(10159):1789–858. doi: 10.1016/S0140-6736(18)32279-7 30496104 PMC6227754

[86] Schoebi D, Campos B. New directions in the psychology of close relationships. London: Routledge; 2019.

[87] Randall AK, Bodenmann G. The role of stress on close relationships and marital satisfaction. Clin Psychol Rev. 2009;29(2):105–15. doi: 10.1016/j.cpr.2008.10.004 19167139

[88] Brisini KSC, Solomon DH. Relational transitions and stress: Turbulence over the lifespan of marriage. The Oxford handbook of the physiology of interpersonal communication. New York, NY, US: Oxford University Press; 2020. p. 97–116.

[89] Segrin C, Flora J. Family communication, 2nd ed. New York, NY, US: Routledge/Taylor & Francis Group; 2011. x, 492–x, p.

[90] Huston TL, Holmes EK. Becoming parents. Handbook of family communication. LEA’s communication series. Mahwah, NJ, US: Lawrence Erlbaum Associates Publishers; 2004. p. 105–33.

[91] Ferber SG, Shoval G, Weller A, Zalsman G. Not one thing at a time: When concomitant multiple stressors produce a transdiagnostic clinical picture. World Journal of Psychiatry. 2023;13(7). 10.5498/wjp.v13.i7.402 PMC10401502 37547732

[92] Cherak SJ, Rosgen BK, Amarbayan M, Wollny K, Doig CJ, Patten SB, et al. Mental Health Interventions to Improve Psychological Outcomes in Informal Caregivers of Critically Ill Patients: A Systematic Review and Meta-Analysis. Crit Care Med. 2021;49(9):1414–26. doi: 10.1097/CCM.0000000000005011 33826586 PMC8373445

[93] Landstedt E, Coffey J. The social context of youth mental health. Routledge; 2017.

[94] Landstedt E, Hammarström A, Winefield H. How well do parental and peer relationships in adolescence predict health in adulthood? Scandinavian Journal of Public Health. 2015;43(5):460–8. doi: 10.1177/1403494815576360 25816863

[95] Rajaleid K, Nummi T, Westerlund H, Virtanen P, Gustafsson PE, Hammarstrom A. Social adversities in adolescence predict unfavourable trajectories of internalized mental health symptoms until middle age: results from the Northern Swedish Cohort. European Journal of Public Health. 2016;26(1):23–9. doi: 10.1093/eurpub/ckv150 26275987

[96] Woodman D. Life out of Synch: How New Patterns of Further Education and the Rise of Precarious Employment Are Reshaping Young People’s Relationships. Sociology-the Journal of the British Sociological Association. 2012;46(6):1074–90.

[97] Haraldsson K, Lindgren EC, Hildingh C, Marklund B. What makes the everyday life of Swedish adolescent girls less stressful: a qualitative analysis. Health Promotion International. 2010;25(2):192–9. doi: 10.1093/heapro/dap061 20233834

[98] Tinnfalt A, Brunnberg E, Ericksson C. Adolescent girls’ and boys’ perceptions of mental health. European Journal of Public Health. 2007;17:140-.

[99] Williams N, Kirkbride JB. Community-based interventions on the social determinants of mental health in the UK: an umbrella review. Journal of Public Mental Health. 2024;ahead-of-print(ahead-of-print).

[100] Rebok GW, Bradshaw CP, Volk HE, Mendelson T, Eaton WW, Letourneau EJ, et al. 285C11Models of Stress and Adapting to Risk: A Life Course, Developmental Perspective. In: Eaton WW, Fallin MD, editors. Public Mental Health: Oxford University Press; 2019. p. 0.

[101] George LK. Life-Course Perspectives on Mental Health. In: Aneshensel CS, Phelan JC, editors. Handbook of the Sociology of Mental Health. Boston, MA: Springer US; 1999. p. 565–83.

