# Supplementary material for: Perceived stress across population segments characterized by differing stressor profiles—A latent class analysis
Source: PLoS One. 2025 Jan 16;20(1):e0316759. doi: 10.1371/journal.pone.0316759 (PMC11737799; doi:10.1371/journal.pone.0316759)
Supplement: S1 File — (DOCX) [file pone.0316759.s001.docx]

# Supporting information: Data availability

Link to website (in Danish): [Hvordan har du det? - DEFACTUM - Social, sundhed og arbejdsmarked](https://www.defactum.dk/forskning/befolkningsundersogelser/hvordan-har-du-det/)

**How to gain access to the dataset**

We invite researchers to apply for analysis rights to the “How are you?” data.

Any granted permission only applies to scientific or statistical research and publication in regular academic journals. Permission does not include any rights to patents or other economic agreements that would lead to financial gain.

Data extraction takes 6 to 10 weeks. If you are applying for data with personal identification numbers (CPR numbers), the extraction may take longer.

**How to Apply**

You must complete the application form for researchers and Ph.D. students, where you describe the purpose of the requested data.

- Make the best use of the data, preferably in interdisciplinary collaborations
- Prevent any potential ambiguities

Link to application form (in Danish): <https://www.defactum.dk/siteassets/indhold---sider/forskning/befolkningsundersogelser/hhdd/adgang-til-data/ansogning_hhdd_videregivelse_forskere-og-kommuner_maj-2022.pdf>

**Guidelines**

To process data access applications as quickly as possible, it is important that applications are complete. Please read the guidelines for managing HHDD data.

Link to guidelines (in Danish): <https://www.defactum.dk/siteassets/indhold---sider/forskning/befolkningsundersogelser/hhdd/adgang-til-data/hhdd-dataadgang---revideret-19-10-2015.pdf>

Note: Access to data from the most recent “How are you?” population health survey is available. However, it is also possible to compare it with data from similar surveys dating back to 2001.

**Contact**

Applications and inquiries to: Hvordanhardudet@rm.dk

You are also welcome to contact a member of the research team if you have additional questions.

If you are sending a letter, please address it to:

DEFACTUM

Attn: Befolkningsundersøgelser

Olof Palmes Allé 15

8200 Aarhus N

Denmark
