# Supplementary material for: Perceived stress across population segments characterized by differing stressor profiles—A latent class analysis
Source: PLoS One. 2025 Jan 16;20(1):e0316759. doi: 10.1371/journal.pone.0316759 (PMC11737799; doi:10.1371/journal.pone.0316759)
Supplement: Attachment — Submitted filename: Review_PONE-D-23-43197.docx [file pone.0316759.s002.docx]

The authors use LCA to describe stressor profiles, their social patterning, and associations with perceived stress levels, among a representative sample of Danish adults. There are important aspects to be addressed; please find some comments below:

1. For the ANOVA and multinomial regression models, authors describe using “procedures developed in recent years to relate latent class membership to external variables”. This is quite vague; please elaborate on the approach used to assign individuals to their best fit class.
2. Missingness is not discussed. Did the 32,417 survey respondents have complete data on all variables of interest? Are the LCA and subsequent analyses based on this full sample of 32,417 respondents? What proportion of respondents was missing and/or excluded from analyses and what measures, if any, were taken to address this? Please discuss.
3. The final selected model has low-class separation and is cause for concern. While substantive interpretation and clinical relevance help justify selection of this 7-class model, additional corroboration using other model parameters would add further support. Each parameter carries its own strengths and limitations to justify its use. Suggest that authors review other information criteria (e.g. consistent Akaike Information Criterion [CAIC], adjusted Bayesian Information Criterion [aBIC]) and/or model indices (e.g., the likelihood-ratio G^2^ statistic) and present this information to lend further support for model selection.
4. Can the authors present results on the mean number of reported perceived stressors for each class? This data is omitted from Table 3 and could add value to the description and discussion of stressor profiles.
5. Results seemingly diverge from theories linking “greater stressor diversity” to better “affective well-being”. Take Class 3, for example, where disease (and deaths) among relatives is the only major stressor and perceived stress levels and effects sizes are lower than the other 6 stressor profiles. Alternatively, consider Class 4, where probabilities are moderate (~0.3) across several stress domains (i.e., work, family, friend and partner relationships, and own disease) but quite high (~0.5) for poor social support, and perceived stress levels and effects sizes are very high. Perhaps better perceived social support is a mitigating factor. Do authors have thoughts on other factors that may promote resilience among this segment (i.e., Class 3) of the Danish population? Please comment.
6. Please review and correct inconsistencies in reporting:
   a. **Abstract:** “Average PSS varied from 9.0 in Class 1 to 24.2 in Class 7 and 26.0 in Class 6.” Figure 1 describes an average PSS of 25.0 for Class 6.
   b. **Results:** “... while 29% were residing with at least one child aged 0-15 years.” Table 1 reports 28% of sample residing with at least one child aged 0-15 years.
   c. **Results:** “... minimal class size of 2% are considered acceptable.” Table 2 reports minimal class size of 3% for 7-class solution.
   d. **Results:** “Marital status: Being married or cohabitating increased the likelihood of belonging to Class 3 and decreased the probability of belonging to Classes 2, 5, 7, and particularly Class 4, compared with Class 1”. Table 4 suggests that these results reflect single status and not being married or cohabitating.
7. Minor grammatical errors:
   a. Omitted word in bold and underlined: “Furthermore, we apply a life-course perspective to the identified segments to enhance our understanding **of** the dynamic interaction between the segments.”
   b. Omitted word in bold and underlined: “Class 4 included 8% of the population with a stressor burden mainly characterized **by** lack of social support and strained relationships”.
